# The usefulness of texture and color enhancement imaging to identify the minor papilla orifice

**DOI:** 10.1002/deo2.358

**Published:** 2024-04-04

**Authors:** Yoshihiro Goda, Kuniyasu Irie, Hideyuki Anan, Yuichi Suzuki, Aya Ikeda, Ryosuke Ikeda, Hiroaki Kaneko, Soichiro Sue, Haruo Miwa, Shin Maeda

**Affiliations:** ^1^ Division of Gastroenterology Yokohama City University School of Medicine Graduate School of Medicine Kanagawa Japan; ^2^ Gastroenterological Center Yokohama City University Medical Center Kanagawa Japan

**Keywords:** endoscopic retrograde cholangiopancreatography, minor papilla, pancreas divisum, pancreatic duct, texture and color enhancement imaging

## Abstract

In clinical cases of pancreas divisum, endoscopic retrograde cholangiopancreatography often necessitates cannulation of the pancreatic duct through the minor papilla. Nevertheless, this procedure can be challenging because of the small size of the minor papilla and the difficulty in visualizing the ductal orifice. A new image‐enhanced endoscopy technique called texture and color enhancement imaging (TXI) has been developed, which enhances texture, brightness, and color compared with white‐light imaging, resulting in subtle differences in the surface mucosa. Herein, we describe the case of a 73‐year‐old man with pancreas divisum in whom TXI was useful in identifying the orifice of the minor papilla. He was referred to our hospital with repetitive acute exacerbation of chronic pancreatitis. Since contrast‐enhanced computed tomography revealed a pancreatic stone in the main pancreatic duct, endoscopic retrograde cholangoepancreatography was performed as a therapeutic intervention. Despite the initial difficulty in identifying the orifice of the minor papilla on white‐light imaging, TXI enhanced its visibility successfully, enabling dorsal pancreatic duct cannulation via the minor papilla. Subsequently, endoscopic pancreatic sphincterotomy was performed and a 6Fr plastic stent was placed. Post‐endoscopic therapy, the patient's abdominal pain was relieved. TXI was useful in identifying the minor papilla orifice and led to successful cannulation.

## INTRODUCTION

In clinical cases of pancreas divisum, the performance of endoscopic retrograde cholangiopancreatography (ERCP) necessitates the cannulation of the pancreatic duct through the minor papilla. The cannulation of the dorsal pancreatic duct via the minor papilla could be challenging because of the small size and inadequate visualization of the ductal orifice.^1^

Texture and color enhancement imaging (TXI; Olympus Medical Systems) is a new image‐enhanced endoscopy technique that enhances texture, brightness, and color compared with white‐light imaging, enabling the detection of subtle differences in the surface mucosa. TXI operates in two distinct modes: Mode 2 accentuates texture and brightness, and Mode 1 adds emphasis on color tone. Although a recent report suggests its utility in identifying the bile duct orifice of the major papilla,^2^ no such reports exist for the orifice of the minor papilla. Here, we present a technique for identifying the orifice of the minor papilla using TXI Mode 1.

## CASE REPORT

**FIGURE 1:**
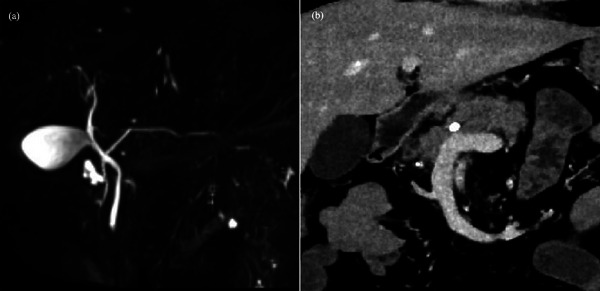
(a) Magnetic resonance cholangiopancreatography showing pancreas divisum. (b) Contrast‐enhanced computed tomography shows a pancreatic stone; the main pancreatic duct does not dilate.

**FIGURE 2:**
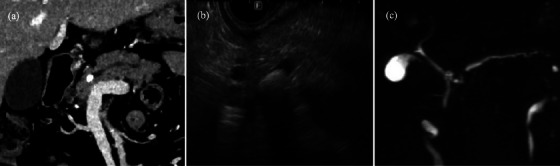
Contrast‐enhanced computed tomography (a), endoscopic ultrasonography (b), and magnetic resonance cholangiopancreatography (c) show a pancreatic stone in the main pancreatic duct and caudal pancreatic duct dilatation.

**FIGURE 3:**
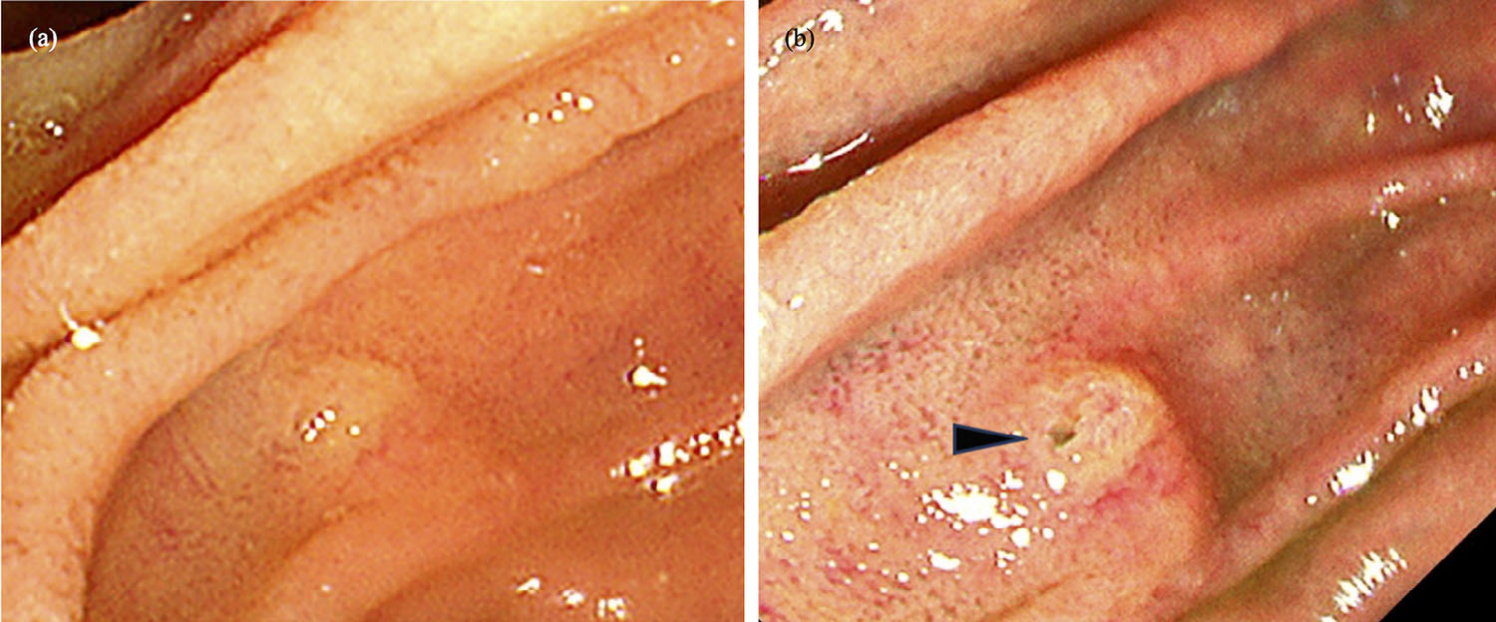
(a) Minor papilla orifice is unclear on white light imaging. (b) Texture and color enhancement imaging Mode 1 enhanced the visibility of the minor papilla orifice (black arrowhead).

**FIGURE 4:**
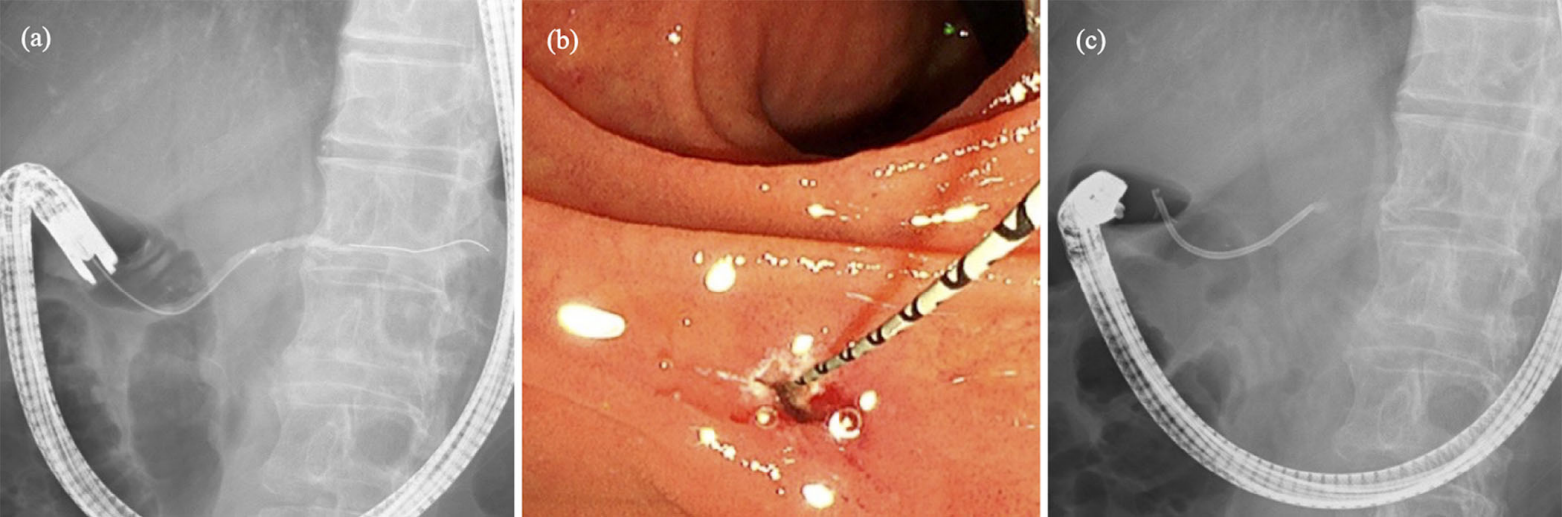
(a) Endoscopic retrograde pancreatography shows no stenosis in the main pancreatic duct. (b) Endoscopic sphincterotomy of the minor papilla was performed. (c) 6Fr Plastic stent was placed.

A 73‐year‐old man with chronic pancreatitis and pancreas divisum (Figure 1) was referred to our hospital with an acute‐onset of abdominal pain. Laboratory studies revealed significantly elevated levels of pancreatic enzymes. Contrast‐enhanced computed tomography detected a stone in the main pancreatic duct of the pancreatic head, which was attributed to be the cause of the symptoms (Figure 2a). The patient was hospitalized with acute exacerbation of chronic pancreatitis. Despite conservative treatment, the patient experienced a recurrence of acute exacerbation of chronic pancreatitis 4 days post‐discharge. Endoscopic ultrasonography and magnetic resonance cholangiopancreatography revealed the presence of a stone in the main pancreatic duct of the pancreatic head without malignancy (Figure 2b,c). Considering the occurrence of relapse within a short period of time, we attempted pancreatic drainage via the minor papilla. A duodenoscope (TJF Q290V; Olympus Medical Systems) reached the second portion of the duodenum, and the endoscopic approach was performed at a long position, in which the duodenoscope was pushed along the greater curvature of the stomach. The minor papilla is located 1‐fold away from the oral side of the major papilla. Attempts to identify the orifice of the minor papilla using white‐light imaging were futile owing to its small size and similar color tone to the surrounding mucosa (Figure 3a). In order to visualize the mucosal surface structure and identify the orifice of the minor papilla, TXI Mode 1 was employed, and the orifice was observed as a dark point (Figure 3b). Dorsal pancreatic duct cannulation via the minor papilla was successfully achieved using a tapered catheter (SHOREN; Kaneka Medix) loaded with a 0.025‐inch guidewire (Fielder; Asahi Intec Co.). Pancreatography revealed no stones or strictures in the main pancreatic duct (Figure 4a), indicating that the stone was located in a branch of the pancreatic duct rather than the main pancreatic duct. The acute exacerbation of chronic pancreatitis is caused by either temporary main pancreatic duct compression by a stone in the branch pancreatic duct, dysfunction of the minor papilla, or dominant duct syndrome.^3^ Endoscopic sphincterotomy of the minor papilla was performed and a 6Fr plastic stent (Harmo‐Ray; Hanaco Medical) was placed (Figure 4b,c). The patient did not experience abdominal pain after the ERCP procedure.

## DISCUSSION

Pancreas divisum, with a prevalence of about 2.9%,^4^ manifests as a congenital malformation resulting from the fusion failure of the ductal systems of the dorsal and ventral pancreatic ducts. Consequently, the ventral duct is short and opens into the major papilla, while the longer dorsal duct opens into the minor papilla with no connection between the ducts. Therefore, cannulation of the dorsal pancreatic duct via the minor papilla becomes necessary when performing ERCP in patients with pancreas divisum.

Dorsal pancreatic duct cannulation of the minor papilla is a challenging procedure. Conti et al reported that the technical success rate of the minor papilla cannulation was 82.1%.^5^ The reasons for the difficulty of the minor papilla cannulation are the inability to identify the minor papilla and the absence of a visible orifice.^1^ Several studies have described the methods for the detection of minor papillary orifices. Devereaux et al. demonstrated the efficacy of synthetic porcine secretin. According to their findings, the administration of synthetic porcine secretin resulted in successful minor papilla cannulation in 25 out of 28 patients (89.8 %) in whom the cannulation was initially unsuccessful. No adverse events associated with synthetic porcine secretin were reported.^6^ Park et al. documented the efficacy of methylene blue spraying on the duodenal mucosa. Among the cohort where initial minor papillary cannulation had failed despite the use of secretin, 11 out of 13 patients had successful cannulation.^7^ While these techniques have been recognized for their utility and efficacy, they require additional materials, and their effectiveness gradually wanes over time. Synthetic porcine secretin is not covered by Japanese national insurance, limiting its practical applicability in clinical settings.

TXI, a new image‐enhancing endoscopy system, has been installed in the EVIS X1 series (Olympus Medical Systems). TXI separates the image components into a structural image, which is an uneven component, and a base image, comprising both brightness and color components. By applying enhancements to each of these images and composing them, the contrast of the unevenness is increased, and the brightness is corrected for bright and dark areas. The resultant images reveal surface irregularities and color variations. The effectiveness of TXI has been frequently reported in detecting and visualizing gastrointestinal diseases.^8^ In the context of pancreatobiliary lesions, TXI has proven to be a valuable tool in identifying anastomotic strictures and bile duct orifices,^2^, ^3^, ^4^, ^5^, ^6^, ^7^, ^8^, ^9^ as well as in enhancing the diagnostic quality of peroral cholangioscopy.^10^ However, there is currently no published evidence of its usefulness in identifying the orifice of the minor papilla. After searching medical literature databases, including PubMed and Web of Science up to the year 2023, using the keywords ‘minor papilla,’ ‘cannulation,’ and ‘TXI,’ we found no reports similar to our study; this suggests that our report presents a novel approach that has not been previously documented in the literature.

In the present case study, TXI Mode 1 was used to successfully locate the minor papillary orifice, which was visualized as a distinct dark point. TXI operates in two distinct modes. Mode 1 incorporates color enhancement beyond Mode 2, intensifying mucosal differences; this heightened tonal contrast proved valuable in identifying the minor papilla orifice. However, caution is advised, as the emphasis in Mode 1 is somewhat pronounced, and prolonged usage may lead to eye fatigue or nausea. It is recommended to turn the TXI on and off as needed judiciously.

The limitation is that TXI cannot enhance the outflow of the pancreatic juice because it is colorless. In general, the minor papilla is located 1‐fold above the major papilla in the 1 o'clock direction.^1^ However, in cases where the location is suspected elsewhere, the outflow of the pancreatic juice from the orifice should be checked to confirm the minor papilla by other method combinations, such as spraying methylene blue, because there is still no evidence for the differentiation of duodenum lesions by TXI. Another limitation is that the use of TXI is limited based on its dependence on the EVIS X1 series system. However, the advantages of TXI include its ease of use, promptness, repeatability, and lack of requirement for additional materials.

In conclusion, our case demonstrates the potential application of TXI in facilitating successful dorsal pancreatic duct cannulation through the identification of the minor papillary orifice. Thus, TXI is a useful technique to improve the success rate of dorsal pancreatic duct cannulation in clinical settings.

## CONFLICT OF INTEREST STATEMENT

None.
